# 'Imported risk' or 'health transition'? Smoking prevalence among ethnic German immigrants from the Former Soviet Union by duration of stay in Germany - analysis of microcensus data

**DOI:** 10.1186/1475-9276-9-15

**Published:** 2010-06-11

**Authors:** Katharina Reiss, Jacob Spallek, Oliver Razum

**Affiliations:** 1 University of Bielefeld, Department of Epidemiology & International Public Health, School of Public Health, P.O. Box 10 01 31, D-33501 Bielefeld, Germany; 2 Bremen Institute for Prevention Research and Social Medicine, Department of Prevention and Evaluation, Unit Social Epidemiology, Linzer Str. 10, 28359 Bremen, Germany

## Abstract

**Background:**

It can be assumed that *resettlers *(ethnic German immigrants from the Former Soviet Union) show similar smoking patterns as persons in their countries of origin at the time of migration. We analysed how the smoking prevalence among *resettlers *differs from that among the general population of Germany and whether the prevalence differs between groups with increasing duration of stay.

**Methods:**

To estimate the smoking prevalence we used the scientific-use-file (n = 477,239) of the German 2005 microcensus, an annual census representing 1% of all German households. Participation in the microcensus is obligatory (unit-nonresponse <7%). We stratified the prevalence of smoking among *resettlers *and the comparison group (population of Germany without *resettlers*) by age, sex, educational level and duration of stay. In total, 14,373 (3% of the total) persons were identified as *resettlers*.

**Results:**

Female *resettlers *with short duration of stay had a significantly lower smoking prevalence than women in the comparison group. With increasing duration of stay their smoking prevalence appears to converge to that of the comparison group (e.g.: high educational level, age group 25-44 years: short duration of stay 15%, long duration of stay 24%, comparison group 28%). In contrast, the smoking prevalence among male *resettlers *with short duration of stay was significantly higher than that among men in the comparison group, but also with a trend towards converging (e.g.: high educational level, age group 25-44 years: short duration of stay 44%, long duration of stay 35%, comparison group 36%). Except for female *resettlers *with short duration of stay, the participants with low educational level had on average a higher smoking prevalence than those with a high educational level.

**Conclusions:**

This is the first study estimating the smoking prevalence among *resettlers *by duration of stay. The results support the hypothesis that *resettlers *brought different smoking habits from their countries of origin shortly after migration. The observed convergence of the smoking habits with increasing duration of stay is in line with the hypothesis of migration as 'health transition'. However, due to the cross-sectional design of the study, further research is needed to confirm these findings.

## Background

Germany is one of the countries with the highest number of immigrants in Europe [1]. In 2007, 15.4 million persons or 18.7% of the entire German population have been identified as having a migrational background; 53% of them have a German nationality, 47% have a foreign nationality [2]. Among the immigrants with German nationality are the so called *resettlers *(German: *Aussiedler *or *Spätaussiedler*), ethnic Germans who returned to Germany from Eastern European countries since the early 1950 s, with the largest number migrating in the mid-1990 s (213,214 in 1994). The total number of *resettlers*, who immigrated to Germany between 1950 and 2005 amounts to 4,481,882 persons, more than half of those coming from Former Soviet Union countries [3].

Compared to other migrant groups, the *resettlers *had a privileged legal situation. With their immigration to Germany and their acceptance as *resettlers*, they gained the legal position as so-called 'Germans by status' and would acquire German citizenship until the 31^st ^of July 1999 by means of a formal naturalisation. Since the 1^st ^August 1999 the legal situation changed and *resettlers *automatically acquired the German citizenship without a formal naturalisation process. Thus, in contrast to other migrant groups, the *resettlers *are Germans by constitution immediately after their migration to Germany. Furthermore, the German law allows persons with a permanent residence in Germany to keep or acquire a foreign citizenship besides the German one upon application.

The analysis of the health situation of migrants is of special interest as they tend to show different health risks and resources as well as health behaviour compared to the population of the host country [4]. However, it can be assumed that their health risks change, both in a positive and negative direction, with increasing duration of stay in the host country [5]. Data concerning morbidity and health behaviour of *resettlers *in Germany is still scarce. With regard to mortality, *resettlers *have a lower all-cause mortality, cardiovascular mortality and cancer mortality compared to the general population of Germany, although there are differences between e.g. type of cancer, age and sex [6-8].

Smoking is known to be an important risk factor for chronic diseases, such as lung cancer or diseases of the cardiovascular system. Analyses of smoking patterns in the countries of the Former Soviet Union reveal that smoking is much more prevalent in men than in women. Bobak et al. (2006) determined a smoking prevalence of 64% among Russian men and 15% among Russian women in 2004 [9]. In Germany, a smoking prevalence of 32% among men and 22% among women has been estimated for 2005 [10]. There is still insufficient data with regard to smoking prevalence among *resettlers *in Germany. On the basis of a cross-sectional study, Aparicio et al. (2005) found no difference in smoking prevalence between *resettlers *and the general population of Germany. In both groups the overall prevalence of smoking was 26%, stratification by duration of stay was not performed [11]. Reeske et al. (2009) furthermore analysed changes in smoking prevalence among Turkish migrants and their offspring in Germany, also on the basis of the German 2005 microcensus [12]. The prevalence of smoking among male Turkish migrants (with high educational level) decreased over the generations, whereas that among female migrants increased. In the second generation, the prevalence partly converged to that among the German reference population or was even higher.

According to Peto et al. (1994), differences in smoking prevalence between countries can be explained by their different position in the stages of the so-called 'smoking epidemic'. The authors developed a model describing the 'smoking epidemic' as a process with four stages. In the 1^st ^stage, smoking is considered as an innovation and is primarily taken up by men. In the 2^nd ^stage, the smoking prevalence among men increases and reaches its peak. In the same stage, women also take up smoking. The 3^rd ^stage is characterised by an increase of the smoking prevalence among women. While it reaches the peak, the smoking prevalence among men starts declining gradually. The 4^th ^stage shows a decline in prevalence among both men and women [13].

A definite attribution of the Former Soviet Union countries and Germany to the respective stages of the model turns out to be complicated. In the countries of the Former Soviet Union, the smoking prevalence among men is higher than in Germany, whereas it is lower among women. Ginter (1995) compared the prevalence of smoking between 15 socialist and 25 democratic countries. The results showed a higher smoking prevalence among men in socialist countries, compared to men in democratic countries. For women the opposite has been observed [14]. Thus, the countries of the Former Soviet Union appear to be in the 2^nd ^stage and Germany in the 3^rd ^stage of the 'smoking epidemic'.

We analysed (I) whether the smoking prevalence among *resettlers *shortly after migration (a proxy for their smoking patterns before migration) differs from that of the general population of Germany; and (II) whether the smoking prevalence among *resettlers *differs between groups with increasing duration of stay (an indication that a 'transition' between the stages of the 'smoking epidemic' has occurred). The underlying hypotheses were (I) that *resettlers *show the same smoking prevalence as the general population of the Former Soviet Union and therefore differ from the general population of Germany shortly after migration; and (II) that *resettlers *move from the early to the later stages of the 'smoking epidemic' after migration and adapt to the smoking prevalence among the general population of Germany with increasing duration of stay.

## Methods

To estimate the smoking prevalence, data from the German 2005 microcensus was used. The microcensus is a census conducted annually including a sample of 1% of all German households (enrolling approx. 380,000 households and 820,000 individuals) [10]. The households have to participate, so the microcensus provides reliable and representative cross-sectional data on Germany's population structure [15]. With about 6% the unit-nonresponse of the 2005 microcensus is low. The item-nonresponse is below 10% for most of the items, depending on whether it is voluntary or obligatory to respond to a certain question [16]. The microcensus consists of an annual basic programme and an additional programme that is included every four years. The basic programme contains questions concerning the demographic, social and economic situation of the participants; the additional programme includes information on health and migration amongst others. While it is voluntary to respond to the health questions, answering the questions concerning migration is obligatory [17]. In 2005, these additional questions on migrational background were added for the first time.

For this study the scientific-use-file of the 2005 microcensus for secondary analyses was used. This file is a randomly drawn, representative sub-sample of the microcensus including data on 477,239 individuals (equating to 70% of the households of the microcensus) [15,16]. The questionnaire did not explicitly ask about the *resettler *status. Thus, to identify the *resettlers*, 20 variables were built (with regard to the actual legal situation of the *resettlers*) to construct only one indicator variable. The reference group was the population of Germany without the *resettlers*. It is not limited to Germans without migrational background as a comparison with the general population of Germany instead of a selected sub-population was intended.

A *resettler *in Germany was categorised as follows: short duration of stay of up to 5 years, medium duration of stay of 6 to 15 years and long duration of stay of 16 or more years due to the fact that the largest number of *resettlers *migrated to Germany between 1986 and 1999 [3]. Concerning the citizenship before naturalisation as well as the existing foreign citizenship the microcensus questionnaire only offered the possibility to choose between Russian or Soviet citizenship in general.

The smoking status of the participants was based on the question "Are you a smoker at present?", which was answered voluntarily. Persons answering "yes, regularly" and "yes, occasionally" were categorised as 'smokers', those stating "no" were categorised as 'non-smokers'. Due to the fact that the number of occasional smokers was very small and no definition of regular and occasional smoker was given in the questionnaire, both smoking types were combined.

For education a dichotomous variable was constructed as well. The inclusion criteria for persons with a high level of education were a graduation from secondary school (*Gymnasium *or *Realschule*) after more than 10 years or a current attendance of grade 11 or higher at the *Gymnasium *as well as any subsequent tertiary education. All those not meeting these criteria have been classified as having a low educational level. A second inclusion criterion for both low and high educational level was an age of at least 18 years. Thus, the final analyses are limited to persons aged 18 years and older.

Data sets of persons with missing data in one of these variables (smoking and education) were excluded from data analysis. The non-response concerning the smoking question was 14% among *resettlers *and 16% among participants from the reference population.

This descriptive analysis estimated smoking prevalence for the *resettlers *with different duration of stay as well as for the reference group, the population of Germany without *resettlers*. As smoking prevalence differs by age and sex and is known to be associated with educational level, results were stratified by these variables to control their influence. To statistically test the differences in smoking prevalence between *resettlers *with different durations of stay and the reference population, the chi-square-test was used (significance level: 0.05). Analyses were performed using SPSS 15.0.

## Results

Within the scientific-use-file of the 2005 microcensus 14,373 *resettlers *have been identified. This is 3% of the total number of individuals in the data set. After excluding persons under the age of 18 years and those with missing data, the total number of *resettlers *amounted to 13,158 *resettlers*, the number of the reference population was reduced by 18% (84,708) to 378,158 persons. Only 9% of the *resettlers *population answered that they migrated to Germany between 2000 and 2005, 42% stated that they had stayed in Germany for 6 to 15 years. Another 43% came to Germany 16 years or more ago and 6% have been identified as *resettlers *without stating any year of arrival. Due to the fact that the majority of the *resettlers *migrated to Germany before 1999, all of those acquired the German citizenship by a formal naturalisation process.

In terms of the smoking prevalence there was no difference between the *resettlers *and the reference population before stratification. In both populations 25% belonged to regular or occasional smokers. After stratification by sex, age and level of education a clear difference in the smoking prevalence was observed between the two comparison groups.

### Women

The prevalence of smoking among female *resettlers *with **high educational level **and **short duration of stay **was significantly lower than that among women from the reference population, independent of age. From those aged 18-24 years 11% of the female migrants with high educational level and short duration of stay and 31% of the women from the comparison group smoked (Table 1). When comparing the smoking prevalence between *resettlers *with short and long duration of stay and the particular comparison group, the following became apparent: The prevalence among female migrants with **high educational level **and **long duration of stay **and that among women from the reference group was similar. Thus, the smoking prevalence among 18-24 year old female *resettlers *with high educational level and long duration of stay was 30%. In the reference population 31% of the women stated to be smokers.

**Table 1:** Smoking prevalence and number of smokers among female *resettlers *and women from the reference population

*Resettlers*												
	**Age in years**	**All ***resettlers*****		**Short duration of stay**		**Medium duration of stay**		**Long duration of stay**		**Reference population**		**p-values^1^**
			
		**%**	**n**	**%**	**n**	**%**	**n**	**%**	**n**	**%**	**n**	

High level of education	18-24	24.1	135	**11.1**	8	**23.2**	69	**30.1**	41	**30.7**	3725	**<0.01**
	
	25-44	19.0	275	**14.6**	19	**15.9**	104	**23.9**	122	**28.3**	10648	**<0.01**
	
	45-64	14.7	131	**8.6**	5	**5.9**	21	**22.5**	99	**22.3**	5874	**<0.01**
	
	≥ 65	4.7	10	0	0	-	-*	5.3	9	8.0	810	0.45
	
	total	17.7	551	**11.9**	32	**14.5**	195	**21.5**	271	**24.4**	21057	**<0.01**

Low level of education	18-24	27.4	68	**14.3**	5	**28.0**	44	**33.3**	12	**47.7**	1421	**<0.01**
	
	25-44	23.5	174	**8.8**	7	**17.2**	66	**40.4**	86	**42.7**	5755	**<0.01**
	
	45-64	14.8	136	**10.8**	8	**7.2**	26	**22.2**	99	**26.3**	7010	**<0.01**
	
	≥ 65	3.9	40	**0**	0	-	-*	**5.7**	36	**5.9**	2227	**<0.01**
	
	total	14.3	418	**8.1**	20	**11.5**	140	**17.6**	233	**20.2**	16413	**<0.01**

Both educational levels combined	18-24	25.2	204	**12.1**	13	**24.9**	114	**30.8**	53	**34.0**	5160	**<0.01**
	
	25-44	20.6	453	**12.2**	26	**16.4**	172	**28.8**	209	**32.1**	16454	**<0.01**
	
	45-64	14.7	267	**9.6**	13	**6.5**	47	**22.3**	198	**24.3**	12926	**<0.01**
	
	≥ 65	4.1	50	**0**	0	**1.5**	5	**5.6**	45	**6.3**	3048	**<0.01**
	
	total	16.1	974	**9.9**	52	**13.2**	338	**19.5**	505	**22.4**	37588	**<0.01**

-* = number 1 to 4

^1^p-values are results of chi-square testing for differences between *resettlers *with different durations of stay and the reference population in each row

Concerning the smoking prevalence among women with low educational level the same trend was observed as for those with a high level of education. The smoking prevalence among female *resettlers *with **low educational level **and **short duration of stay **also differed from that among women from the reference population. Furthermore, the prevalence among female *resettlers *with **low educational level **and **long duration of stay **and the particular reference group was similar again. Nevertheless, a difference between high and low level of education was observed for the *resettlers*, independent of age and duration of stay (Table 1). The smoking prevalence among *resettlers *with high educational level was slightly lower than that among *resettlers *with low educational level. Concerning the reference group a difference between high and low educational levels became apparent as well. Independent of age, the women with a high level of education had a lower smoking prevalence than those with a low one. This difference between high and low educational level was more distinctive for women from the reference population than for female *resettlers*.

### Men

In contrast to female *resettlers*, the smoking prevalence among male *resettlers *with **high educational level **and **short duration of stay **was significantly higher than that among the reference group, also independent of age. From those in the age group 24-44 years, 44% of the male migrants with high level of education and short duration of stay reported to be smokers in contrast to 36% of the men from the comparison group (Table 2). Similar smoking prevalences in those with **high educational level **and **long duration of stay **and the particular reference group were also seen in men. The smoking prevalence among 25-44 year old male *resettlers *with high educational level and long duration of stay was 35%. In the reference population 36% of the men stated to be smokers. As among women, this trend in smoking prevalence by duration of stay was observed among men with **low educational level**.

**Table 2:** Smoking prevalence and number of smokers among male *resettlers *and men from the reference population

*Resettlers*												
	**Age in years**	**All *resettlers***		**Short duration of stay**		**Medium duration of stay**		**Long duration of stay**		**Reference population**		**p-values^1^**
			
		**%**	**n**	**%**	**n**	**%**	**n**	**%**	**n**	**%**	**n**	

High level of education	18-24	32.0	123	40.4	19	31.2	58	30.0	36	35.1	3848	0.52
	
	25-44	38.3	425	**44.2**	42	**41.6**	200	**34.7**	149	**35.5**	12239	**0.02**
	
	45-64	30.8	239	**37.9**	22	**36.0**	105	**25.0**	100	**28.7**	7520	**<0.01**
	
	≥ 65	12.8	22	-	-*	-	-*	12.8	17	10.6	1111	0.86
	
	total	33.1	809	**41.0**	84	**37.1**	366	**27.9**	302	**30.1**	24718	**<0.01**

Low level of education	18-24	52.1	174	50.0	23	53.8	121	48.9	23	58.2	2645	0.22
	
	25-44	52.6	478	**63.0**	63	**53.4**	241	**46.3**	133	**51.8**	8725	**0.04**
	
	45-64	41.8	391	**50.0**	33	**44.2**	168	**39.6**	180	**35.7**	8923	**<0.01**
	
	≥ 65	13.2	88	-	-*	12.0	22	13.5	60	14.3	3478	0.89
	
	total	39.7	1131	**50.8**	123	**44.6**	552	**32.1**	396	**33.6**	23771	**<0.01**

Both educational levels combined	18-24	41.4	298	44.7	42	43.6	179	35.7	60	41.9	6518	0.40
	
	25-44	44.7	906	**54.0**	107	**47.1**	442	**39.3**	282	**40.9**	21024	**<0.01**
	
	45-64	36.9	636	**44.0**	55	**40.9**	277	**32.8**	282	**32.1**	16510	**<0.01**
	
	≥ 65	13.2	111	14.3	5	11.8	25	13.5	78	13.2	4619	0.95
	
	total	36.7	1951	**46.2**	209	**41.3**	923	**30.2**	702	**31.7**	48671	**<0.01**

-* = number 1 to 4

^1^p-values are results of chi-square testing for differences between *resettlers *with different durations of stay and the reference population in each row

Also, the smoking prevalence among male *resettlers *with low educational level was higher than the prevalence among those with a high level of education, independent of age and duration of stay (high education: 44% of those aged 25-44 years with short duration of stay, low education: 63% of those aged 25-44 years with short duration of stay) (Table 2). In the reference group the difference between high and low educational was observed as well. In contrast to the women, the difference between high and low educational level was distinctive for both men from the reference population and male *resettlers*.

In terms of the difference in smoking prevalence between women and men it became apparent that the prevalence among male *resettlers *was much higher than that among female *resettlers*, independent of age, educational level and duration of stay. In the age group 25-44 years, the smoking prevalence among male *resettlers *with medium duration of stay and high educational level was e.g. up to 3-times as high as that among female *resettlers *(42% to 16%). The smoking prevalence among men from the reference population is also higher than that among women, although the difference between the sexes is not as large as for *resettlers*.

## Discussion

**Figure 1:**
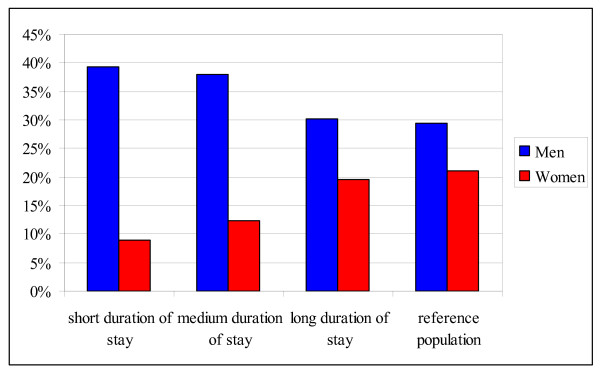
**Smoking prevalence among *resettlers *and the reference population by sex and duration of stay (n = 190,064)**.

The smoking prevalence among female *resettlers *appears to increase with increasing duration of stay and converges to that among women from the comparison group. In contrast to that, it seems that the smoking prevalence among male *resettlers *decreased with increasing duration of stay, thereby also converging towards the prevalence among men from the comparison group (Figure 1).

These differences between *resettlers *with short duration of stay and the comparison group are in line with findings from former studies. Kyobutungi et al. (2006) and Becher et al. (2007) found higher mortality rates from lung cancer - as one of the most important health outcomes caused by smoking - for male *resettlers *compared to native-born German men and lower mortality rates for female *resettlers *compared to native-born German women. As possible reasons the authors state that *resettlers *are subject to the same exposures as persons in their countries of origin and therefore e.g. also show similar smoking patterns shortly after migration [6,7]. Therefore, these findings allow the conclusion that shortly after migration the *resettlers *virtually 'import' the smoking patterns and thereby the related health risks from their countries of origin.

This may also be the reason for the differences in smoking prevalence between male and female *resettlers *with short duration of stay. McKee et al. (1998) and Gilmore et al. (2004) stated a higher proportion of smokers among men from Former Soviet Union countries compared to women [18,19]. Also in this study the prevalence among male *resettlers *exceeds that among females, independent of age, educational level and duration of stay.

McKee et al. (1998) also showed that Russian men and women with low educational level (as measured by secondary, tertiary and vocational education) more often stated to be smokers than those with high educational level [18]. Whereas this can be applied to male *resettlers *in this study and both men and women from the reference population, it is not applicable to female *resettlers *with short duration of stay. In this group a clear difference between high and low level of education was not observed. One possible explanation for this finding could be conservative values and norms among *resettlers *[20]. Thus, smoking among women would be considered as inappropriate or even unacceptable, independent of educational level [21].

**Figure 2:**
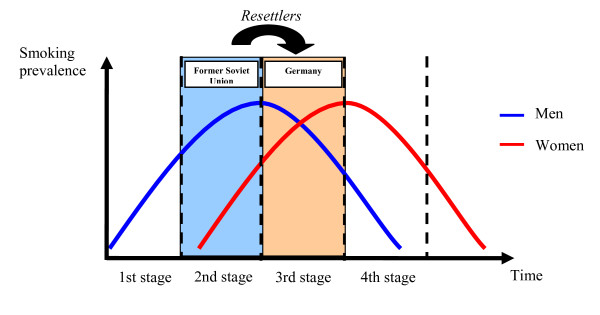
**Movement of the *resettlers *between the stages of the 'smoking epidemic' as a 'health transition' (adapted from Peto et al. 1994 **[13]).

Another important result of this study is that the smoking prevalence among *resettlers *with long duration of stay appears to converge to that among the general German population. On the one hand, the countries of origin and the host country of the *resettlers *have different positions in the 'smoking epidemic'. On the other hand, the smoking prevalence between *resettlers *with short and long duration of stay differs. Whereas the already mentioned 'imported risk' becomes apparent among *resettlers *with short duration of stay, this risk seems to virtually disappear with increasing duration of stay. One possible explanation is the 'health transition' migrants experience when they migrate, in this case in terms of the 'smoking epidemic'. Since most non-migrant populations are also gradually undergoing a health transition, it is more appropriate to use the term '*accelerated *health transition' for this phenomenon [5]. Hence, in the course of the immigration the *resettlers *move from the second stage (the position of the countries of origin) to the third stage (the position of the host country) of the epidemic (Figure 2). With increasing duration of stay a convergence to the smoking patterns of the host country takes place. This explanation is also supported by the findings of Ködderitzsch (1997). The author describes the *resettlers*' strong 'willingness to integrate', leading to an adaptation to the behavioural patterns of the host country. Thus, the migration to Germany is rather considered as 'remigration', meaning a 'return' to the home country of the ancestors [22].

Whether these findings can be generalised to other migrant groups in Germany or whether the smoking prevalence among *resettlers *converges to the general German population because they are ethnic German immigrants, cannot be said. According to Reeske et al. (2009) the smoking prevalence among the offspring of Turkish migrants in Germany also partly converged to that among the German reference population or even exceeded it [12].

The strength of this study is the size and representativeness of the German 2005 microcensus, being the largest annual cross-sectional survey in Europe with more than 800,000 persons interviewed [10]. As a result, representative statements concerning the smoking prevalence among *resettlers *as well as among the reference population can be made. Due to the additional questions on migration it is possible to identify the *resettlers *as migrants with German nationality on the basis of their year of arrival, nationality, date of naturalisation and nationality before naturalisation. Another important strength is that the effect of the duration of stay on the smoking prevalence among *resettlers *could be analysed for the first time.

The main limitations of this study are due to the design of the 2005 microcensus. Since the survey provides cross-sectional (but not longitudinal) data, it is not possible to speak of a 'time trend' in terms of the smoking prevalence [15]. In this study it was not possible to observe changes in smoking habits among the same group of *resettlers *over years but merely to compare the smoking habits among those who had a short, medium and long duration of stay in 2005. Hence, there is still the possibility that the smoking prevalence among *resettlers *with medium and long duration of stay merely reflects different smoking patterns in the countries of origin before migration. These might have been similar to those of their future host country. In other words: There is still the possibility that a 'cohort effect' occurred. As that is the major weakness of this study, data on smoking trends in Russia in the early 1990 s was acquired. Perlman et al. (2007) state that between 1992 and 2003, smoking prevalence among both women and men in Russia increased from 6.9% to 14.8% and from 57.4% to 62.6% respectively. The large gap in prevalence between men and women in 1992 and the increase over time indicate that Russia was still in the 2^nd ^stage of the 'smoking epidemic' in the early 1990 s [23]. Moreover, Hearn et al. (1991) claim that the smoking pattern seen in the 1980 s in the Former Soviet Union is similar to that in the USA in the late 1960 s [24]. These findings show that among *resettlers *with medium and long duration of stay the smoking patterns in the countries of origin before migration have not been similar to those of their future host country in 2005.

Furthermore, it could be assumed that the prevalence among *resettlers *with long duration of stay results from a high proportion of offspring (often so called 'second generation' migrants), who already show similar behavioural patterns as the general population of Germany. However, only 384 persons (3% of the *resettlers*) have been identified as *resettlers *of the 'second generation', so this explanation is highly unlikely. Nevertheless, a sensitivity analysis (leaving the 'second generation' migrants out) revealed no differences in smoking prevalence stratified by sex, age, educational level and duration of stay. In fact, the number of *resettlers *in this final analysis only reduced by 9 persons, as it is rather unlikely that a *resettler *born in Germany would state a length of stay. However, *resettlers *aged 18-24 years living in Germany for 16 years or more (long duration of stay) have lived most of their lives in their host country. Thus, the smoking behaviour of this group is assumed to be more influenced by their host country than by their country of origin. As the number of this group is rather small (n = 113), their influence on the results is not believed to be major.

In the age group 65 years and older the number of smokers was very low in the *resettlers *population. Thus, the respective data could not be interpreted.

Moreover, since the survey is carried out on a household level, a 'cluster effect' cannot be ruled out. Thus, it is possible that individual survey participants living in the same household also state similar smoking habits and the estimated smoking prevalence in the strata might be slightly too low.

## Conclusions

In spite of some limitations, in particular the cross-sectional design, the findings from this study support the hypothesis that the migrant group of *resettlers *undergo a 'health transition' in terms of the 'smoking epidemic'. Longitudinal studies are needed to further test this hypothesis. Furthermore, a need for preventive action can be derived from the results. Female *resettlers *in particular should be targeted by respective measures in order to prevent their smoking prevalence from rising with increasing duration of stay. The reduction in smoking prevalence among male *resettlers *should also be supported further by adequate actions.

## Competing interests

The authors declare that they have no competing interests.

## Authors' contributions

OR developed the concept of migration as 'health transition'. JS and OR conceived the study. KR, JS and OR carried out the study. KR performed the data analysis and drafted the manuscript. JS participated in the data analysis and coordinated the study. All authors read and approved the final manuscript.
